# Glucagon infusion alters the circulating metabolome and urine amino acid excretion in dogs

**DOI:** 10.1530/JOE-24-0051

**Published:** 2024-06-27

**Authors:** Michael Merkhassine, Reilly W Coch, Carol E Frederick, Lucinda L Bennett, Seth A Peng, Benjamin Morse, Bethany P Cummings, John P Loftus

**Affiliations:** 1 Loftus Laboratory, Department of Clinical Sciences, Cornell University, College of Veterinary Medicine, Ithaca, New York, USA; 2 VCA Colonial Animal Hospital, Ithaca, New York, USA; 3 Weill Cornell College of Medicine, New York, New York, USA; 4 Fate Therapeutics, San Diego, California, USA; 5 Center for Alimentary and Metabolic Science, Department of Surgery, School of Medicine, University of California, Davis, Sacramento, California, USA; 6 Department of Molecular Biosciences, School of Veterinary Medicine, University of California, Davis, Davis, California, USA

**Keywords:** glucagon, amino acid, metabolomics, metabolome, canine

## Abstract

Glucagon plays a central role in amino acid (AA) homeostasis. The dog is an established model of glucagon biology, and recently, metabolomic changes in people associated with glucagon infusions have been reported. Glucagon also has effects on the kidney; however, changes in urinary AA concentrations associated with glucagon remain under investigation. Therefore, we aimed to fill these gaps in the canine model by determining the effects of glucagon on the canine plasma metabolome and measuring urine AA concentrations. Employing two constant rate glucagon infusions (CRI) – low-dose (CRI-LO: 3 ng/kg/min) and high-dose (CRI-HI: 50 ng/kg/min) on five research beagles, we monitored interstitial glucose and conducted untargeted liquid chromatography–tandem mass spectrometry (LC-MS/MS) on plasma samples and urine AA concentrations collected pre- and post-infusion. The CRI-HI induced a transient glucose peak (90–120 min), returning near baseline by infusion end, while only the CRI-LO resulted in 372 significantly altered plasma metabolites, primarily reductions (333). Similarly, CRI-HI affected 414 metabolites, with 369 reductions, evidenced by distinct clustering post-infusion via data reduction (PCA and sPLS-DA). CRI-HI notably decreased circulating AA levels, impacting various AA-related and energy-generating metabolic pathways. Urine analysis revealed increased 3-methyl-l-histidine and glutamine, and decreased alanine concentrations post-infusion. These findings demonstrate glucagon’s glucose-independent modulation of the canine plasma metabolome and highlight the dog’s relevance as a translational model for glucagon biology. Understanding these effects contributes to managing dysregulated glucagon conditions and informs treatments impacting glucagon homeostasis.

## Introduction

Glucagon is a pancreatic endocrine hormone involved in regulating circulating glucose and amino acid (AA) levels and is an important therapeutic target for various metabolic and gastrointestinal conditions (Unger & Cherrington 2012, Sandoval & D’Alessio 2015). Increasing blood glucose concentrations is perhaps the best-known function of glucagon, but its relationship with AAs is increasingly recognized (Liljenquist *et al.* 1981, Boden *et al.* 1984, 1990, Allenspach *et al.* 2000, Larger *et al.* 2016, Wewer Albrechtsen *et al.* 2016, Dean *et al.* 2017, Holst *et al.* 2017, Kim *et al.* 2017, Kraft *et al.* 2017, Galsgaard *et al.* 2018, Hayashi & Seino 2018, Elmelund *et al.* 2022, Richter *et al.* 2022). Most AAs directly stimulate glucagon secretion from the α-cell to various degrees (Assan *et al.* 1977, Bertrand *et al.* 1993, Oya *et al.* 2013, Maruszczak *et al.* 2022) and, in the dog, asparagine appears to be the most potent AA glucagon secretagogue (Rocha *et al.* 1972). It has recently been shown that AAs also indirectly stimulate insulin secretion by a paracrine mechanism involving glucagon action on the β-cell (Capozzi *et al.* 2019). Interestingly, multiple groups have shown that AAs form a feedback loop with glucagon, where increased glucagon decreases plasma AAs through a cognate G protein-coupled glucagon receptor in the liver, resulting the oxidation of AAs into glucose and urea (Dean *et al.* 2017, Holst *et al.* 2017, Galsgaard *et al.* 2018, Richter *et al.* 2022). Expression of the glucagon receptor in the small intestine, brain, pancreatic β-cells, and kidney reflects the importance of glucagon for regulating metabolic pathways of diverse cell types. Proximal renal tubular cells express the glucagon receptor, and glucagon stimulates tubular glucose reabsorption (Marks *et al.* 2003), and reduced renal glucagon receptor expression alters systemic metabolic homeostasis, including increased plasma AA (Wang *et al.* 2024). This suggests that glucagon could have other effects on proximal renal tubular cells, including modulating transport and tubular reabsorption of AAs. Increased urinary AA clearance in a patient with a glucagonoma supports this assertion (Almdal *et al.* 1990). Evaluating renal AA loss in a high glucagon state would provide valuable mechanistic insight into glucagon biology and help influence future nutritional strategies.

Directly, glucagon is most commonly used as an antidote for hypoglycemia caused by exogenous insulin administration (Datte *et al.* 2016, Harris *et al.* 2020). Elevated glucagon levels are seen in virtually all forms of diabetes mellitus (DM), α-cell hyperplasia, and, most dramatically, glucagonoma syndrome (Chastain 2001, Hædersdal *et al.* 2023). Superficial necrolytic dermatitis is a disfiguring skin condition that is characteristic of the glucagonoma syndrome in humans, and a related condition is also present in dogs. Aminoaciduric canine hypoaminoacidemic hepatopathy syndrome (also known as hepatocutaneous syndrome) is the most common cause of superficial necrolytic dermatitis in dogs (Loftus *et al.* 2022), and glucagon has been speculated to play a role in this condition, although we recently reported lower plasma glucagon concentrations in dogs with this syndrome (Holm *et al.* 2023). Glucagon has also been evaluated for diagnostic purposes (Fall *et al.* 2008, Greenbaum *et al.* 2008) and as a metric for carbohydrate metabolism in nutritionally supplemented exercising dogs (Frye *et al.* 2017).

As AAs play critical roles in protein synthesis and metabolic pathways, an enhanced understanding of AA metabolism has broad implications. Most experiments investigating the fate of AAs due to glucagon constant rate infusions (CRIs) found that glucagon reduces plasma AAs by increasing gluconeogenesis from AAs and accelerating AA uptake by the liver (Boden *et al.* 1984, 1990). Investigating the urinary fate of AAs after a glucagon infusion improves our understanding of AA metabolism in hyperglucagonemic states, such as many forms of diabetes. An improved understanding of metabolic changes downstream of glucagon excess could inform the use of drugs that modify glucagon in canine DM and other conditions.

The biological effects of supraphysiologic glucagon concentrations remain underexplored, and the dog provides an excellent translational model for glucagon biology. Previous studies employed pancreatic clamp techniques, where combinations of insulin, glucose, or somatostatin are administered to control for hyperglycemia or isolate the role of glucagon in hepatic glucose production (Shulman *et al.* 1978). A study isolating the effects of severe hyperglucagonemia on glucose and AA metabolism is lacking.

We aimed to address two critical gaps in glucagon biology with broad applications for canine health and foundations for translational research. First was the lack of metabolomic data in dogs as a translational model of glucagon biology. Secondly, glucagon effects on the kidney warrant further explanation, and changes in urine amino acid concentrations in response to exogenous glucagon are lacking. Therefore, we performed a study investigating the effects of two unclamped (i.e. glucose concentrations uncontrolled by insulin or other hormones) glucagon CRIs: a ‘physiological’ dose that achieves circulating concentrations sufficient to engage the hepatic glucagon receptor signaling, and a ‘hyperphysiological’ dose that simulates pathologic concentrations, such as those encountered in the glucagonoma syndrome (Wermers *et al.* 1996). We hypothesized that exogenous glucagon infusion would reduce AAs and related metabolites in the plasma and increase AAs in the urine of dogs. We tested this hypothesis by investigating (i) plasma metabolomic alterations and (ii) urine AA concentrations in response to exogenous glucagon infusion in healthy dogs.

## Materials and methods

### Animals

Five male healthy, purpose-bred research beagles were obtained for this study. Each dog had a complete blood count, chemistry profile, and physical examination to ensure they were apparently healthy before study inclusion. The Cornell University Institutional Animal Care and Use Committee approved this protocol (2020-0083). Continuous interstitial glucose monitoring devices (FreeStyle Libre Pro, Abbott), previously validated in dogs with 99% accuracy for normal and high blood glucose concentrations, were placed as previously described (Corradini *et al.* 2016, Malerba *et al.* 2020). Briefly, a small area (approximately 5 cm × 5 cm) was clipped and aseptically prepared along each dog’s neck. A single sensor was affixed using the pre-packaged adhesive and a small amount of tissue adhesive (3M VetBond Tissue Adhesive). Three dogs dislodged or removed the sensor during the study period and required sensor replacement on the contralateral side. Dogs were normally fed once daily in the morning. They were fasted on the morning of, and throughout, the experiments. A venous sampling catheter (20 g × 20 cm, Drum Long Line Catheter, MILA International, Inc., Florence, KY, USA) was placed in a lateral saphenous vein of each dog. Each dog had an indwelling urinary catheter (6F Foley, MILA International) placed.

### Glucagon infusions

**Figure 1:**
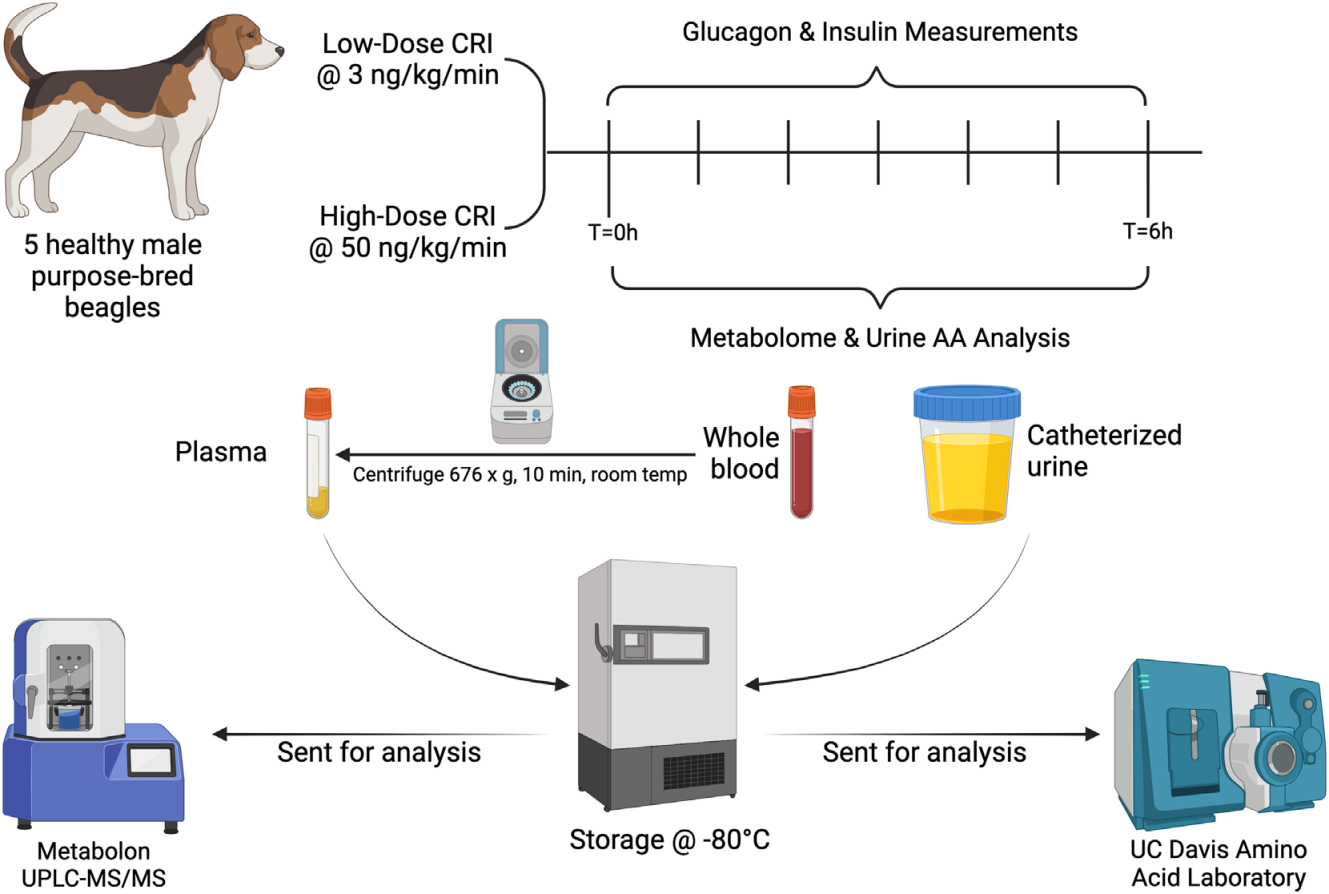
An overview of the experimental design. Five healthy purpose-bred research beagles received low-dose (3 ng/kg/min) and high-dose (50 ng/kg/min) glucagon CRIs over a 6-h period. Blood and urine samples were collected pre-infusion, hourly during the infusion, and post-infusion. Plasma and urine from pre- and post-infusion were then submitted for metabolomic profiling and amino acid analysis. Created with Biorender.com.

Two separate glucagon (Glucagon Emergency Kit, Glucagon for Injection, 1 mg/mL, Amphastar Pharmaceuticals, Inc., Rancho Cucamonga, CA, USA) infusions were performed to compare responses to different glucagon loads. Doses for the CRIs were adapted from previous publications (Liljenquist *et al.* 1981, Boden *et al.* 1984, Datte *et al.* 2016, Pedersen *et al.* 2020). We first conducted the low-dose CRI (CRI-LO, 3 ng/kg/min), which was designed to activate the hepatic GCGR (Pedersen *et al.* 2020). One week later, with no interventions to dogs in between, the high-dose CRI (CRI-HI, 50 ng/kg/min) was conducted, which was intended to simulate a pathologic hyperglucagonemic state (Liljenquist *et al.* 1981). One dog received the entire 6-h dose as a bolus over approximately 15 min due to a pump error and was included in glucose and metabolomic analyses as a serendipitous opportunity to evaluate persistent metabolic changes from a single, high dose of glucagon. However, based on plasma metabolome results, that dog’s results were excluded from urine AA analysis. Both CRIs were delivered over 6 h. During the CRI, the dogs were assessed hourly, including measuring heart and respiratory rates and monitoring for any gastrointestinal signs (e.g. vomiting, nausea, diarrhea). Heparinized whole blood samples (4 mL, BD Vacutainer) were collected pre-infusion, hourly during the infusion, and at 6 h (post-infusion). Whole blood samples were centrifuged at 676 × ***g*** for 10 min at room temperature, yielding platelet-enriched plasma, which was stored at −80°C. Urine samples were also obtained for the same time points and stored at −80°C (Fig. 1). Plasma metabolomic and urine AA analyses were conducted on pre-infusion and 6-h timepoint samples.

### Serum glucagon and insulin measurements

Serum glucagon and insulin were measured on samples collected at hourly intervals as described above by the Endocrinology Laboratory at the Animal Health Diagnostic Center (Cornell University, Ithaca, NY, USA). Insulin was measured by radioimmunoassay for non-equine species, routinely offered for diagnostic testing by the lab. Serum glucagon was measured using a commercially available glucagon ELISA (10-1281-01, Mercodia, Uppsala, Sweden), according to manufacturer instructions, as previously described (Holm *et al.* 2023).

### Amino acid profiles

Urine samples were submitted to the UC Davis Amino Acid Laboratory for AA profiling. This included catheterized urine samples from each dog at the pre-infusion and post-infusion time points for the high-dose infusion. The Amino Acid Laboratory adds 6% sulfosalicylic acid (1:1) to each sample for deproteinization before processing. Urine creatinine was measured enzymatically by the New York State Veterinary Diagnostic Laboratory with a commercial chemistry analyzer (Roche Cobas c501).

### Liquid chromatography–tandem mass spectrometry (LC-MS/MS) analysis and metabolomics profile

Metabolon (Metabolon, Inc, Morrisville, NC, USA) conducted metabolomic profiling on plasma samples as previously described (Evans *et al.* 2009, DeHaven *et al.* 2010, Miller *et al.* 2015, Gillenwater *et al.* 2020). Samples were prepared using the automated MicroLab STAR system from Hamilton Company. Proteins were precipitated with methanol under vigorous shaking for 2 min (Glen Mills GenoGrinder 2000), followed by centrifugation to remove proteins, dissociate small molecules bound to proteins or trapped in the precipitated protein matrix, and recover chemically diverse metabolites. The resulting extract was divided into five fractions: two for analysis by two separate reverse phases (RP)/ultra-performance liquid chromatography (UPLC)-MS/MS methods with positive ion mode electrospray ionization (ESI), one for analysis by RP/UPLC-MS/MS with negative ion mode ESI, one for analysis by HILIC/UPLC-MS/MS with negative ion mode ESI, and one sample was reserved for backup. Samples were placed briefly on a TurboVap (Zymark) to remove the organic solvent. The sample extracts were stored overnight under nitrogen before preparation for analysis.

All methods utilized a Waters ACQUITY UPLC and a Thermo Scientific Q-Exactive high-resolution/accurate mass spectrometer interfaced with a heated electrospray ionization source and Orbitrap mass analyzer operated at 35,000 mass resolution.

The informatics system consisted of four major components, the Laboratory Information Management System, the data extraction and peak-identification software, data processing tools for QC and compound identification, and a collection of information interpretation and visualization tools for use by data analysts. These informatics components’ hardware and software foundations were the LAN backbone and a database server running Oracle 10.2.0.1 Enterprise Edition.

Metabolon maintains a library based on authenticated standards that contain the retention time/index (RI), the mass-to-charge ratio (m/z), and chromatographic data (including MS/MS spectral data) on all molecules present in the library. Furthermore, biochemical identifications are based on three criteria: retention index within a narrow RI window of the proposed identification, accurate mass match to the library ±10 ppm, and the MS/MS forward and reverse scores between the experimental data and authentic standards. The MS/MS scores are based on comparing the ions present in the observed spectrum to those in the library spectrum. While there may be similarities between these molecules based on one of these factors, all three data points can be used to distinguish and differentiate biochemicals.

The Human Metabolome Database (https://hmdb.ca/) was searched for reference information on selected metabolites.

### Statistical analysis

Descriptive statistics are reported as medians and ranges. Due to the sample size, we did not conduct normality testing and applied non-parametric statistics. Differences in interstitial glucose measurements were assessed by the Kruskall–Wallis test with Dunn’s multiple comparisons (time points compared to baseline, with adjusted *P* values). We compared urine amino concentrations by two-way ANOVA and Sidak’s multiple comparisons test (adjusted *P* values). Commercial software (Prism 9.0 or later, GraphPad, RRID:SCR_002798) computed the statistical analyses and generated corresponding graphs. Metabolon’s initial standard statistical analyses were performed in ArrayStudio/Jupyter Notebook on log-transformed data. For those analyses not standard in ArrayStudio/Jupyter Notebook, the programs R (https://cran.r-project.org/) or JMP were used. The matched-pairs *t*-test compared pre- to post-infusion results for each infusion.

Statistical analyses and data visualization of the LC-MS/MS data were performed with MetaboAnalyst 5.0 (RRID:SCR_015539, https://genap.metaboanalyst.ca/), similar to those previously described (Loftus *et al.* 2023). Normalized data from named metabolites (Supplementary File 1, see section on supplementary materials given at the end of this article) were log-transformed (base-10), and Pareto data scaling was applied. Raw *P* values were reported unless otherwise specified. Analysis parameters were set at the software default unless otherwise specified. One dog that received its glucagon dose as a bolus was excluded from some analyses, as declared in the results.

Principal component analysis (PCA) and the sparse partial least squares-discriminant analysis (sPLS-DA) method (one-factor statistical analysis) were chosen for data reduction analysis (Lê Cao *et al.* 2011). Settings were as follows: number of components = 5, variables per component = keep the same number (10). Validation method = five-fold CV (two-component sPLS-DA error rate was 15%). Random forest analysis: number of trees = 500, number of predictors = 7, randomness = on. Heat map analysis was conducted using default settings.

Fold-change and *t*-tests (one-factor statistical analysis) were conducted to identify metabolites significantly increased >two-fold and depicted by volcano plots. The *P*-value threshold was set at a 0.05 false discovery rate. Random forest analysis (RFA) was conducted using default settings.

We conducted enrichment analysis using the over-representation analysis (globaltest method) (Goeman *et al.* 2004) using the Small Molecule Pathway Database. The significant metabolites identified by the fold-change and *T*-tests were used. All compounds in the library were selected for the reference metabolome.

A *P* value of <0.05 established significance.

## Results

### Serum glucagon and insulin concentration increases were dose-dependent

**Figure 2:**
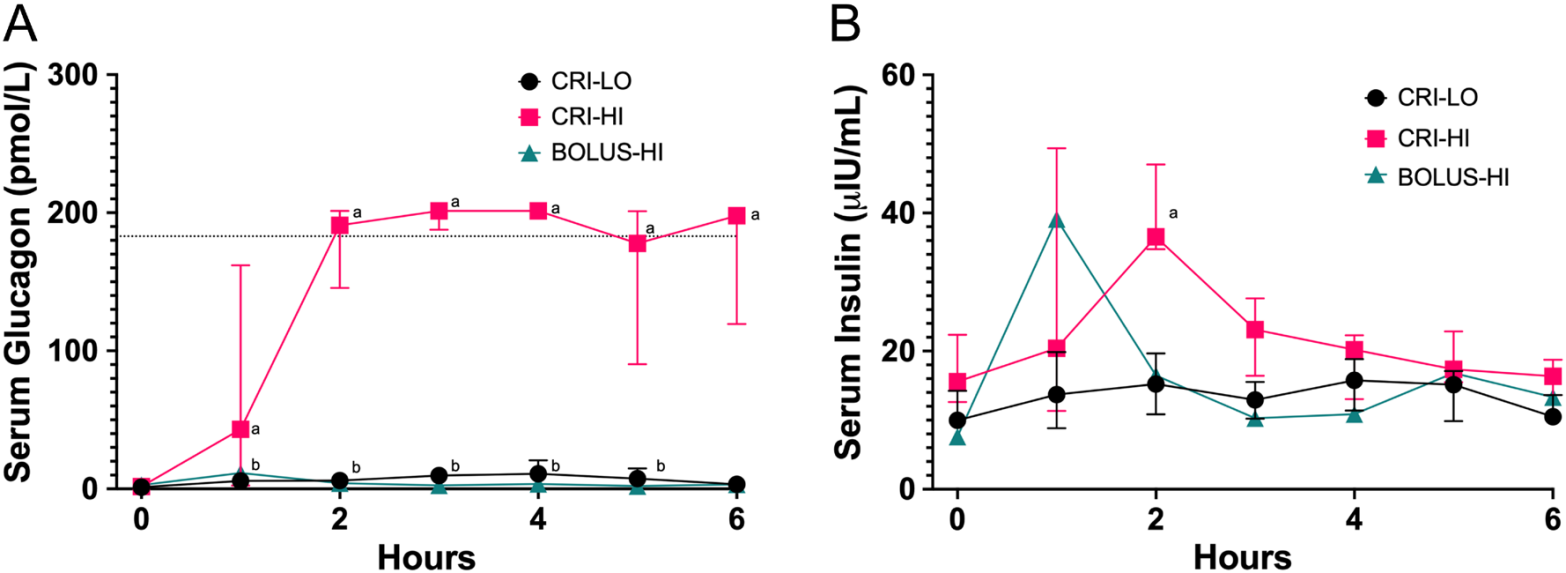
Serum glucagon and insulin measurements of dogs (*n* = 5) administered glucagon constant rate infusions. Serum glucagon (A) and insulin (B) concentrations were measured immediately before and hourly during the infusion. The dotted line in (A) indicates the upper range of the assay sensitivity, thus, values were estimated from the standard curve. Values are medians with error bars depicting the interquartile range. Statistical notations (‘a’ and ‘b’) above data points indicate significantly higher concentrations than the corresponding time-0 for the corresponding CRI group, i.e. high-dose (‘a’ = 50 ng/kg/min, CRI-HI) and low-dose (‘b’ = 3 ng/kg/min, CRI-LO) infusions.

We assessed serum glucagon levels to verify the concentrations achieved with both low- and high-dose infusions. The low-dose infusion resulted in a mild increase, whereas the high-dose infusion led to a substantial elevation in circulating glucagon concentrations. Throughout the observation period, serum glucagon levels were significantly elevated compared to baseline in both experiments, except at the 6-h mark with the low-dose infusion (Fig. 2A). Notably, in dogs receiving the high-dose infusion over 6 h, glucagon concentrations remained elevated consistently. Conversely, when administered as a bolus in one dog over approximately 20 min, glucagon concentrations were comparable to baseline levels at 1–6 h (Fig. 2A).

Furthermore, we examined serum insulin concentrations to provide insights into glucose levels. Serum insulin peaked around 2 h (120 min), with notable increases observed solely in the high-dose glucagon group (Fig. 2B).

### A high dose of exogenous glucagon is required to affect glucose concentrations

**Figure 3:**
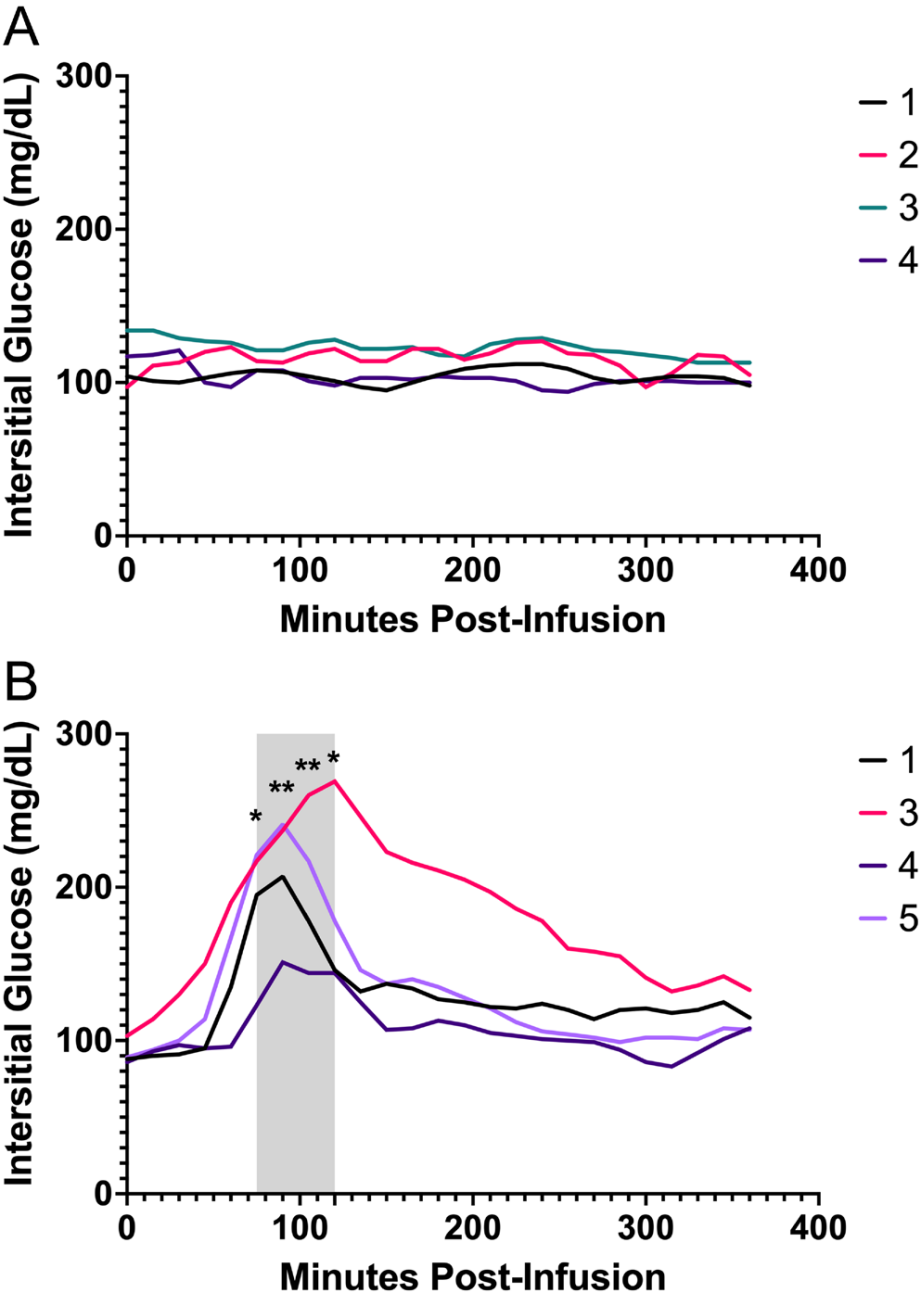
Interstitial glucose (IG) readings via flash glucose monitoring system during glucagon constant-rate infusions. Glucose measurements from the (A) low-dose (3 ng/kg/min) and the (B) high-dose (50 ng/kg/min) glucagon infusions. Each line indicates an individual dog, corresponding to dog numbers in the legend. The shaded area indicates time points where IG was significantly greater than time-0. *P < 0.05, *P < 0.01.

To assess the glycemic effects of a glucagon infusion, interstitial glucose concentrations were obtained for four dogs during each infusion. Glucose concentrations were unaffected during the low-dose glucagon infusion (Fig. 3). In contrast, a transient increase (peak 90–120 min) in glucose occurred during the high-dose infusion, corresponding to insulin increases. Glucose levels returned to near baseline by the end of the 6-h infusion.

### Both doses of exogenous glucagon affect the canine plasma metabolome

**Figure 4:**
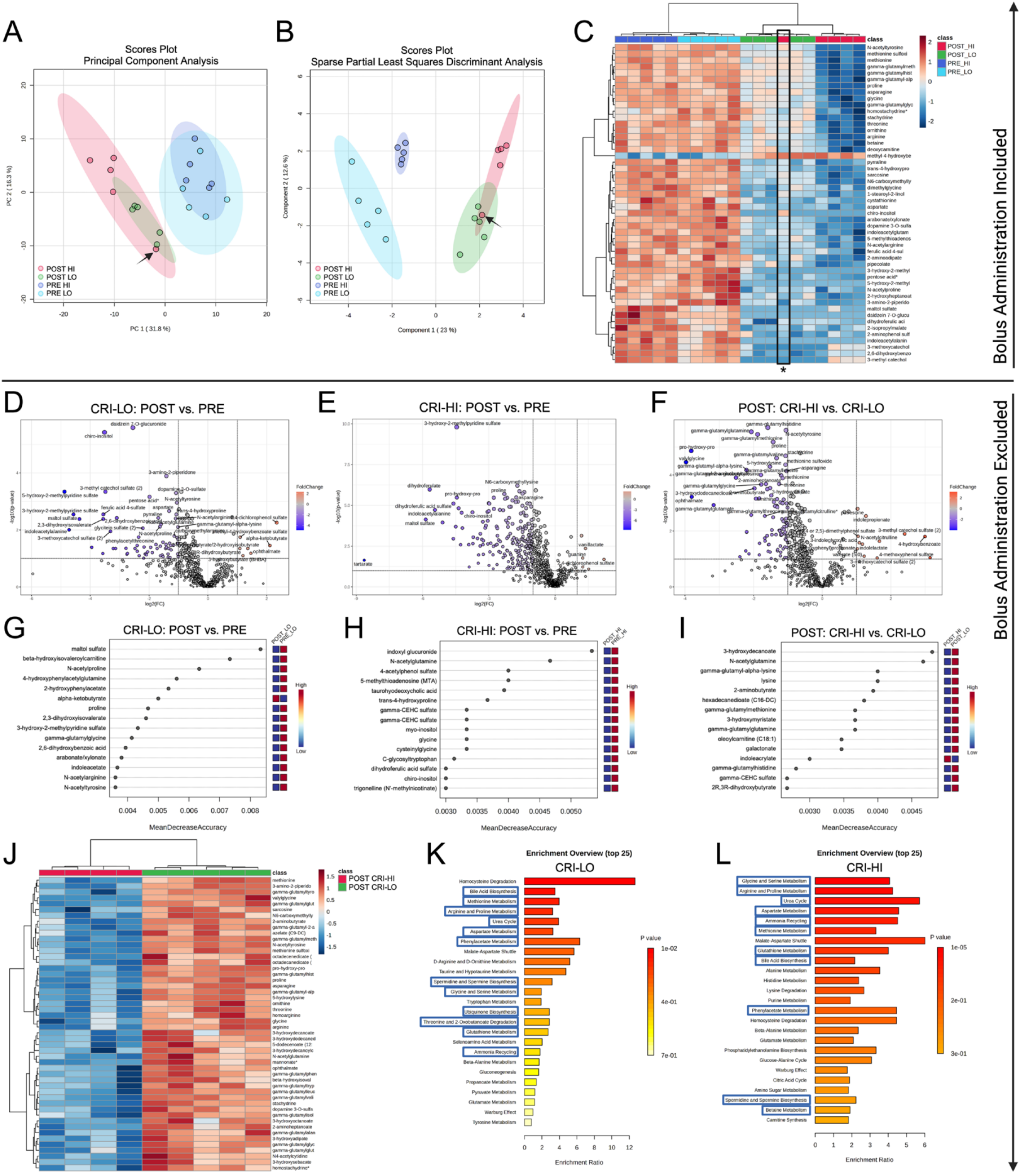
Metabolomic analyses of dogs administered exogenous glucagon infusions. Global multivariate metabolomic analyses of dogs (*n* = 5) administered exogenous glucagon (A–C). Principal component (A) and sparse partial least squares discriminant (B) analyses of plasma metabolites before (PRE) and after (POST) glucagon infusions. (C) Heat map of the top (lowest *P* value) 50 metabolites PRE and after POST LO and 50 ng/kg/min (HI) glucagon infusions. The arrows (A) and asterisk (B) indicate one dog (#2) that received the 50 ng/kg glucagon infusion as an i.v. bolus over 10 min due to a pump error. Metabolomic analyses of dogs (*n* = 5 CRI-LO, *n* = 4 CRI-HI) administered glucagon as a constant rate infusion (CRI) excluding dog number 2 (D–L). Volcano plots (D–F) indicating significant metabolite changes POST glucagon CRI-LO (D) or CRI-HI (E) and POST CRI-HI compared to CRI-LO (F). Metabolites significantly (*P* < 0.05) increased (red) or decreased (blue) greater than two-fold, depicted by single dots. Random forest analysis (RFA) plots (G–I) identified metabolites that best fit a model for distinguishing groups. The RFA models establish metabolites for distinguishing POST glucagon CRI-LO (G) or CRI-HI (H) and POST CRI-LO to CRI-HI (I). Heat map (J) of the top (lowest *P* value) 50 metabolites comparing POST CRI-LO to POST CRI-HI. Pathway enrichment (over-representation) analysis (K,L). The top 25 small molecule pathway database pathways with over-represented significant plasma metabolites in CRI-LO (K) and CRI-HI (L) glucagon infusions are displayed and ranked by *P* value. Pathways boxed in blue indicate pathways also enriched when POST CRI-HI was compared to POST CRI-LO. Created with BioRender.com. CRI-LO/LO = 3 ng/kg/min glucagon infusion. CRI-HI/HI = 50 ng/kg/min glucagon infusion.

We employed an untargeted metabolomics approach to comprehensively assess metabolic changes driven by exogenous glucagon administration in dogs. The low-dose glucagon infusion resulted in significant changes in 372 plasma metabolites, with 333 metabolites reduced compared to baseline (Supplementary Table 1). After the high-dose infusion, 414 metabolite levels significantly changed compared to baseline, with 369 metabolites reduced (Supplementary Table 1). We observed distinct clustering of groups of metabolites by both data-reduction approaches of PCA (Fig. 4A) and sPLS-DA (Fig. 4B), as well as cluster analysis (Fig. 4C), except for the one dog in the CRI-HI group that received the dose as a bolus. This dog clustered with the CRI-LO group and was excluded from subsequent analyses. Generally, metabolite changes were in the same direction for the CRI-LO (Fig. 4D) and CRI-HI (Fig. 4E). Metabolite changes were also more robust in the CRI-HI group than in the CRI-LO group (Fig. 4F). Exogenous glucagon broadly reduced circulating levels of most AA levels (Table 1). Several monoacylglycerols and diacylglycerols were also decreased significantly after glucagon infusions (Table 2), with the monoacylglycerols 1-myristoylglycerol, 1-palmitoleoylglycerol, and 1-oleoylglycerol reduced to less than 20% of pre-infusion concentrations after the CRI-HI. RFA out-of-bag error rates determined by RFA were 0.0 and for CRI-LO, CRI-HI, and comparing CRI-HI to CRI-LO. Random forest analysis identified maltol sulfate as the metabolite that best distinguished the hyperglucagonemic state (Fig. 4G) induced by CRI-LO, and indoxyl glucuronide was the best-distinguishing metabolite in the CRI-HI experiment (Fig. 4H). Maltol sulfate is classified as a xenobiotic, and indoxyl glucuronide is a product of hepatic glucuronidation. Evaluating post-infusion data, lower 3-hydroxydecanoate, a beta-oxidation intermediate, in the CRI-HI group was the best model predictor for discrimination from the CRI-LO. Cluster analysis comparing post-infusion CRI-HI to CRI-LO metabolomes (Fig. 4J) illustrated excellent discrimination between the two doses, with substantial reductions in the top 50 metabolites defining the CRI-HI metabolome compared to the CRI-LO. Pathway enrichment analysis corroborated a predominant impact of glucagon on metabolic pathways related to AA biosynthesis, degradation, or pathways with AAs as critical intermediates such as the urea cycle (Fig. 4K and L). In most cases, metabolites were significantly decreased in affected pathways, for example, all significant changes in urea cycle metabolites were reductions compared to baseline. Generally, the CRI-LO had a lesser impact on metabolic pathways (Fig. 4K and Supplementary Fig. 1) than the CRI-HI (Fig. 4L and Supplementary Fig. 2); however, the CRI-LO had a greater impact on the homocysteine degradation pathway than the CRI-HI. Other notable pathways included bile acid biosynthesis (Table 3), the glucose-alanine cycle, the malate-aspartate shuttle, purine metabolism (Table 4), the citric acid cycle, and carnitine synthesis. Additionally, when comparing CRI-HI to CRI-LO, all but two significant pathways were shared with the enriched pathways identified when CRI groups were compared to their respective baseline (Fig. 4K and L). The two unique pathways identified by comparing POST CRI-HI to CRI-LO were the nicotinate and nicotinamide metabolism and porphyrin metabolism pathways.

**Table 1:** Fold changes in plasma amino acids measured by liquid chromatography tandem mass spectrometry analyzed by the matched pairs *t*-test.

Amino acid	Low-dose glucagon infusion			High-dose glucagon infusion		
	Fold change	*P*-value	*q*-value	Fold change	*P*-value	*q*-value
Arginine	0.71^a^	0.0052	0.0119	0.45^a^	0.005	0.0155
Proline	0.58^a^	0.0044	0.0111	0.29^a^	0.001	0.0096
Carnosine	0.74^a^	0.0228	0.0248	0.53^a^	0.0121	0.0234
3-methyl-L-histidine	0.91	0.2648	0.1232	0.74^a^	0.0244	0.0349
Tryptophan	0.91	0.1118	0.0684	1.03	0.9273	0.4488
Histidine	0.95	0.396	0.1662	0.61^a^	0.0055	0.0163
1-Methylhistidine	1.04	0.3436	0.1497	0.81^a^	0.0783	0.0772
Lysine	0.81^a^	0.0107	0.017	0.48^a^	0.0099	0.0217
Ornithine	0.60^a^	0.0052	0.0118	0.29^a^	0.0018	0.0104
Phenylalanine	1.07	0.4332	0.176	1	0.9431	0.4507
Tyrosine	0.86	0.2432	0.1168	0.64	0.1115	0.099
Leucine	1.18^a^	0.0866	0.0575	0.99	0.8222	0.4162
Isoleucine	1.28^a^	0.0087	0.0156	1.2	0.1799	0.1421
Methionine	0.79^a^	0.0191	0.0225	0.69	0.0066	0.0171
Cysteine	0.9	0.6157	0.2192	0.54^a^	0.0207	0.0317
Valine	1.20^a^	0.0698	0.0507	0.87	0.3189	0.2206
Citrulline	0.82^a^	0.0088	0.0156	0.54^a^	0.006	0.0166
Alanine	0.51^a^	0.0182	0.0221	0.30^a^	0.0019	0.0104
Glycine	0.70^a^	0.0215	0.024	0.43^a^	0.004	0.0145
Glutamine	0.98	0.6834	0.2359	0.57^a^	0.0115	0.0231
Glutamic Acid	0.69*	0.0172	0.0213	0.50^a^	0.0083	0.0191
Asparagine	0.8	0.0681	0.0503	0.42^a^	0.0012	0.0101
Serine	0.72^a^	0.01	0.0164	0.46^a^	0.0015	0.0102
Threonine	0.65^a^	0.0033	0.0095	0.32^a^	0.0024	0.0115
Aspartic acid	0.40^a^	0.0007	0.0074	0.40^a^	0.0123	0.0235
Taurine	0.55^a^	0.001	0.0074	0.48^a^	0.0015	0.0102

^a^ *P* < 0.05.

**Table 2:** Fold changes in plasma monoacylglycerol and diacylglycerols measured by liquid chromatography–tandem mass spectrometry analyzed by the matched pairs *t*-test.

Lipid	Low-dose glucagon infusion			High-dose glucagon infusion		
	Fold change	*P*-value	*q*-value	Fold change	*P*-value	*q*-value
1-Myristoylglycerol (14:0)	0.14^a^	0.0067	0.0133	0.10^a^	0.0008	0.0093
1-Palmitoleoylglycerol (16:1)^b^	0.25	0.1147	0.0693	0.09^a^	0.0009	0.0096
1-Oleoylglycerol (18:1)	0.32^a^	0.0321	0.0312	0.14^a^	0.0009	0.0096
1-Linoleoylglycerol (18:2)	0.35	0.0956	0.0614	0.20^a^	0.0023	0.0115
1-Arachidonylglycerol (20:4)	1.55	0.0685	0.0503	1.18	0.976	0.4584
1-Docosahexaenoylglycerol (22:6)	1.78	0.0758	0.0527	1.54	0.4649	0.2925
2-Oleoylglycerol (18:1)	0.39	0.0917	0.0596	0.32^a^	0.0008	0.0096
2-Arachidonoylglycerol (20:4)	1.48	0.1485	0.0827	1.54	0.0686	0.0699
Palmitoyl-linoleoyl-glycerol (16:0/18:2) [1]^b^	0.73	0.2539	0.1205	0.29^a^	0.0014	0.0102
Palmitoyl-linoleoyl-glycerol (16:0/18:2) [2]^b^	0.57^a^	0.0072	0.0139	0.31^a^	0.0048	0.0155
Palmitoyl-arachidonoyl-glycerol (16:0/20:4) [1]^b^	0.53	0.0971	0.0621	0.58	0.0567	0.0623
Palmitoyl-arachidonoyl-glycerol (16:0/20:4) [2]^b^	0.63^a^	0.0365	0.0334	0.52^a^	0.0042	0.0149
Palmitoleoyl-arachidonoyl-glycerol (16:1/20:4) [2]^b^	0.40^a^	0.0112	0.0173	0.35^a^	0.0201	0.0315
Oleoyl-linoleoyl-glycerol (18:1/18:2) [1]	0.50^a^	0.002	0.0083	0.34^a^	0.0116	0.0231
Oleoyl-linoleoyl-glycerol (18:1/18:2) [2]	0.49^a^	0.0009	0.0074	0.32^a^	0.0162	0.0278
Linoleoyl-linoleoyl-glycerol (18:2/18:2) [1]^b^	0.51^a^	0.0188	0.0224	0.30^a^	0.0232	0.034
Linoleoyl-linoleoyl-glycerol (18:2/18:2) [2]^b^	0.54^a^	0.0062	0.0131	0.34^a^	0.0135	0.025
Linoleoyl-linolenoyl-glycerol (18:2/18:3) [2]^b^	0.53^a^	0.0045	0.0111	0.29^a^	0.004	0.0145
Stearoyl-arachidonoyl-glycerol (18:0/20:4) [1]^b^	1.14	0.216	0.1085	1.19	0.3159	0.2192
Stearoyl-arachidonoyl-glycerol (18:0/20:4) [2]^b^	1.21^a^	0.0402	0.0361	1.33	0.1205	0.1052
Oleoyl-arachidonoyl-glycerol (18:1/20:4) [1]^b^	0.63	0.0807	0.0544	0.6	0.0929	0.0877
Oleoyl-arachidonoyl-glycerol (18:1/20:4) [2]^b^	0.7	0.1241	0.0736	0.50^a^	0.0124	0.0236
Linoleoyl-arachidonoyl-glycerol (18:2/20:4) [1]^b^	0.52	0.1188	0.0711	0.48	0.1957	0.1517
Linoleoyl-arachidonoyl-glycerol (18:2/20:4) [2]^b^	0.74	0.204	0.1044	0.43^a^	0.0128	0.0241
Linoleoyl-docosahexaenoyl-glycerol (18:2/22:6) [1]^b^	1.47	0.2702	0.1246	1.03	0.8762	0.4341
Linoleoyl-docosahexaenoyl-glycerol (18:2/22:6) [2]^b^	1.46	0.052	0.0419	0.83	0.3881	0.2551

Brackets with numbers indicate the presence of isomers of other compounds in the spectral library.

^a^ *P* < 0.05.

^b^Indicates a compound that has not been confirmed based on a standard, but metabolon is confident in its identity.

### Urine amino acid concentrations remain stable despite reduced plasma concentrations

**Figure 5:**
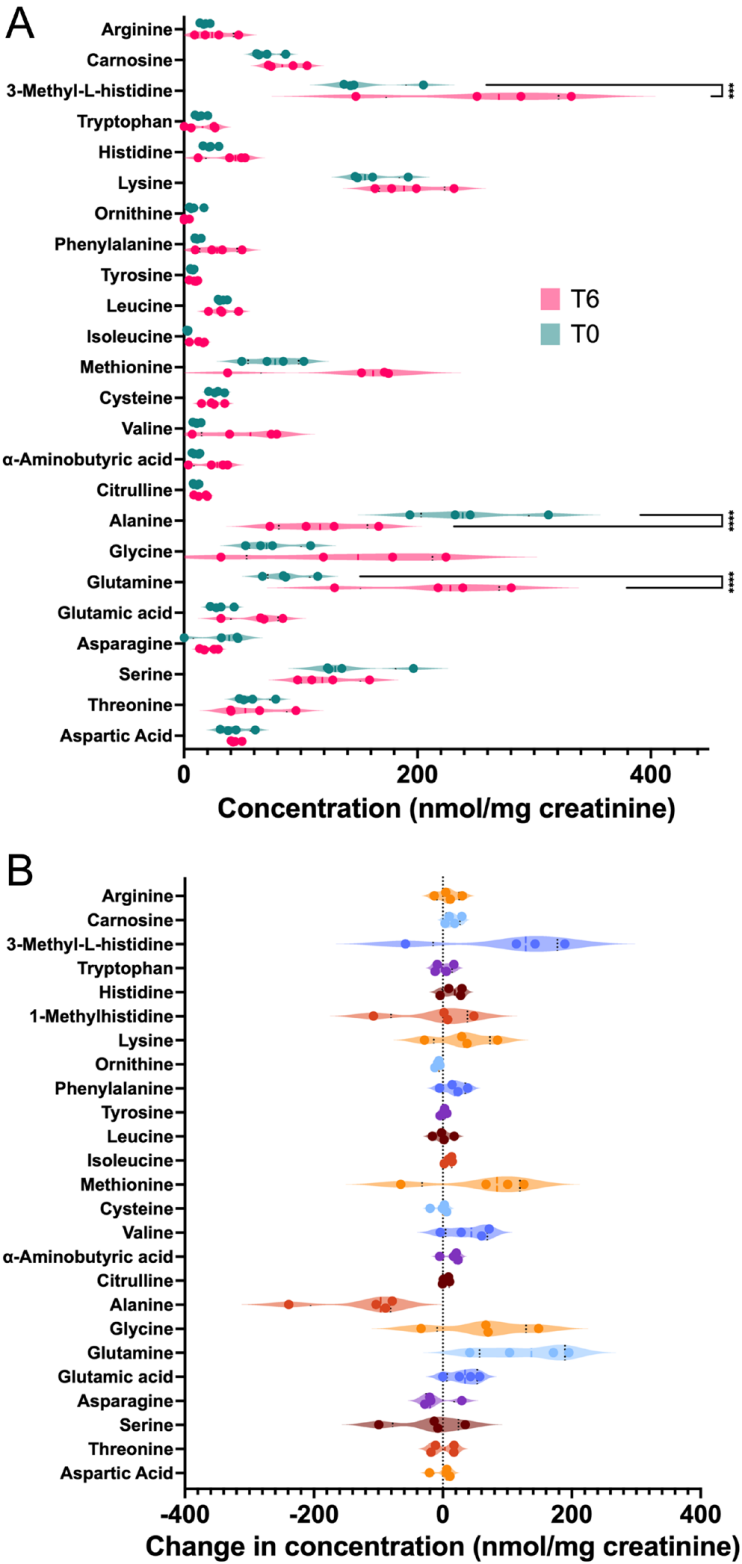
Changes in creatinine-normalized urine amino acid concentrations after a 6-h glucagon infusion. Violin plots indicating the effects on urine amino acid concentrations following the high-dose (50 ng/kg/min) glucagon infusion. Measured absolute amino acid concentrations (A) and concentration changes (B) in dogs before (T0, green) and after (T6, pink) the infusion. Dots indicate individual dog data points. Dashed lines indicate medians and dotted black lines indicate quartiles. Values for 1-methylhisitidine were removed from (A) for clarity. ***P < 0.001, ****P < 0.0001.

We aimed to gain insight into the alterations in urine AA concentrations associated with glucagon excess. Given the results of our metabolomic analysis, we excluded the AA results from the dog that received its glucagon dose as a bolus. Despite semi-quantitatively lower plasma levels (Table 1), urine concentrations of glutamine and 3-methylhistidine were significantly increased, while alanine concentrations were reduced (Fig. 5). Although not significantly increased, lysine, phenylalanine, methionine, valine, glycine, and glutamic acid all showed positive median changes post-infusion.

## Discussion

Our study aimed to investigate changes in the metabolome of dogs administered exogenous glucagon at supraphysiologic concentrations. Secondarily, we assessed renal AA handling by quantitative measurement in the urine. Glucagon broadly impacted the canine plasma metabolome, with profound reductions in circulating AA and acylglycerol levels. In contrast, urine AAs were not broadly reduced and, in some cases, were significantly increased, suggesting glucagon directly or indirectly affects renal AA handling.

Concomitant with increasing glucagon levels, serum insulin concentrations peaked approximately 2 h post-infusion in the CRI-HI group, an appropriate response to hyperglycemia that explains the normalization of glucose concentrations. A recent study investigated glucose concentrations in response to hyperglucagonemia and also noted transient hyperglycemia in an insulin-clamped canine model (Coate *et al.* 2024). Comparisons between CRI-HI and CRI-LO groups suggest glucagon’s predominant influence on AA regulation over glucose homeostasis, evidenced by minimal to no impact on insulin and glucose concentrations in the CRI-LO group. A serendipitous observation from a dog receiving a high-dose bolus of glucagon revealed substantial alterations in the serum metabolome even six hours post-administration, highlighting glucagon’s primary role in modulating AA metabolism. Employing a continuous glucose monitor was a novel approach, given its limited use in dogs beyond diabetic management studies (Re *et al.* 2023). However, its use in canine studies beyond device validation or use in diabetic dogs has been limited to one study evaluating the effect of a ghrelin receptor agonist on glycemic control (Pascutti *et al.* 2022). While blood glucagon concentrations can vary, those achieved in our study align with a hyperglucagonemic state. Although interstitial glucose monitoring was occasionally interrupted, consistent trends in glucose excursions across dogs and parallels with human studies reinforce conclusions regarding supraphysiological glucagon’s glycemic effects.

The essential role of glucagon in systemic AA homeostasis is increasingly recognized (Cahill & Aoki 1977). Experiments demonstrating glucagon’s effect on circulating AA concentrations have been conducted in humans and animal models (Bromer & Chance 1969, Landau & Lugibihl 1969, Boden *et al.* 1984, 1990, Couet *et al.* 1990, Flakoll *et al.* 1994). More recently, the plasma metabolome of non-diabetic, overweight, and obese people in response to glucagon infusion was described (Vega *et al.* 2021). Similar to the findings in our study, Vega *et al.* found that infusions of glucagon most prominently affected AA and lipid pathways. Although the study used similar infusions of glucagon, the human subjects were infused for a much longer period (72 h) and included a placebo cohort. In contrast, we compared 6-h samples to pre-infusion samples. Nevertheless, the similarities in results are compelling and further support the dog as an appropriate comparative model for glucagon physiology. Characterizing the effects of glucagon excess on the canine plasma metabolome is pivotal, ensuring that the canine model continues to be a relevant contributor to advancing our understanding of glucagon biology.

Reduced plasma AA concentrations in response to glucagon have primarily been attributed to the uptake and oxidation of AAs in the liver, providing an important substrate for hepatic glucose production via gluconeogenesis. This phenomenon was described in the dog, where the net hepatic uptake of many AAs was increased in response to glucagon infusion, resulting in a 17% decrease in circulating AA levels and coinciding with a 30% increase in hepatic glucose production (Flakoll *et al.* 1994). Nitrogen liberated by AA catabolism is primarily eliminated by the urea (ornithine) cycle, reflected by increased excretion of total urinary nitrogen (Almdal *et al.* 1990). Similar to the results in humans who received high-dose glucagon infusion (Vega *et al.* 2021), our study found significantly reduced concentrations of ornithine citrulline and urea in the plasma. Although we did not measure total nitrogen in our urine samples, we speculate that these changes represented enhanced nitrogen turnover and elimination induced by the combined actions of glucagon in the liver and kidney. Another notable finding was that branched-chain amino acids were mildly increased in the CRI-LO group but reduced in response to the CRI-HI conditions. Interestingly, increased branched-chain amino acids can promote insulin resistance (Newgard 2012) and thus may be related to insulin resistance associated with mild increases in glucagon.

Glucagon-induced changes in the canine plasma lipidome were also similar to those seen in humans (Vega *et al.* 2021), with diacylglycerols significantly decreased. We also noted substantial monoacylglycerol reductions, which were not documented in the human study. These findings align with increased β-oxidation reported in rodents and humans in hyperglucagonemic states (Guettet *et al.* 1988, Xiao *et al.* 2011). Other pathways affected in our study included purine metabolism and bile acid synthesis. The impact of glucagon on plasma purines (Table 4) in our study may be attributable to increased hepatic *de novo* purine synthesis, as previously reported in a rat model (Itakura *et al.* 1987). Primary and secondary bile acids were globally decreased (Table 3). These reductions may be related to glucagon’s choleretic effect or changes in enterohepatic recirculation (Guettet *et al.* 1988, Kaminski *et al.* 1988).

**Table 3:** Fold changes in plasma bile acids measured by liquid chromatography–tandem mass spectrometry analyzed by the matched pairs *t*-test.

Bile acid	Low-dose glucagon infusion			High-dose glucagon infusion		
	Fold change	*P*-value	*q*-value	Fold change	*P*-value	*q*-value
Cholate	0.13	0.0623	0.0475	0.24^a^	0.0488	0.0566
Taurocholate	0.37	0.1416	0.0807	0.24^a^	0.0027	0.0122
Taurochenodeoxycholate	0.48	0.131	0.0766	0.15^a^	0.0019	0.0104
β-Muricholate	0.11^a^	0.0066	0.0133	0.22^a^	0.0013	0.0101
α-Muricholate	0.14^a^	0.0046	0.0111	0.22^a^	0.0005	0.0091
Tauro-β-muricholate	0.26	0.074	0.0521	0.16^a^	0.0044	0.0154
Deoxycholate	0.23	0.0702	0.0509	0.38	0.0844	0.0815
Taurodeoxycholate	0.39	0.1357	0.0786	0.10^a^	0.0009	0.0096
Lithocholate	1	–	–	1	–	–
Taurolithocholate	0.25	0.0509	0.0415	0.07^a^	0.0004	0.0086
Ursodeoxycholate	0.14^a^	0.0063	0.0131	0.18^a^	0.0021	0.0109
Tauroursodeoxycholate	0.32	0.0707	0.051	0.19^a^	0.0074	0.0182
Taurohyodeoxycholic acid	0.29^a^	0.0013	0.0074	0.27^a^	0.0099	0.0217
12-Dehydrocholate	0.13^a^	0.0386	0.0352	0.25^a^	0.0099	0.0217
7-Ketodeoxycholate	0.11^a^	0.0188	0.0224	0.24^a^	0.0059	0.0166
Ursocholate	0.26	0.0664	0.0499	0.49^a^	0.0204	0.0316

^a^ *P* < 0.05.

**Table 4:** Fold changes in plasma purines measured by liquid chromatography–tandem mass spectrometry analyzed by the matched pairs *t*-test.

Purine metabolite	Low-dose glucagon infusion			High-dose glucagon infusion		
	Fold change	*P*-value	*q*-value	Fold change	*P*-value	*q*-value
Inosine	0.87	0.7714	0.253	1.94^a^	0.0024	0.0115
Hypoxanthine	0.91	0.9261	0.2864	1.71	0.1581	0.1287
Xanthine	0.65	0.4488	0.1799	0.99	0.9881	0.4608
Xanthosine	0.54^a^	0.0111	0.0173	0.61	0.0873	0.0835
N1-methylinosine	0.99	0.994	0.3003	0.81	0.4837	0.3006
Urate	0.67^a^	0.0046	0.0111	0.95	0.5877	0.3398
Allantoin	0.79^a^	0.0023	0.0083	0.68^a^	0.0146	0.0261
Allantoic acid	0.82^a^	0.0066	0.0133	0.73	0.0599	0.064
1-Methylhypoxanthine	0.74	0.2394	0.1157	0.66	0.0536	0.0603
Adenosine 5′-monophosphate	1.22	0.5287	0.1993	1.63	0.1194	0.1044
Adenosine	1.07	0.6616	0.23	1.9	0.0573	0.0626
Adenine	0.95	0.7647	0.252	0.8	0.1549	0.1264
N1-methyladenosine	0.92^a^	0.0382	0.0349	0.96	0.6518	0.3604
N6-carbamoylthreonyladenosine	0.85	0.0631	0.0481	0.72^a^	0.0302	0.0409
N6-succinyladenosine	0.99	0.6466	0.226	0.92	0.4729	0.2968
Guanosine	0.66	0.7964	0.2592	2.93^a^	0.0078	0.0184
Guanine	1.05	0.5186	0.1974	2.82^a^	0.0039	0.0145
7-Methylguanine	0.73^a^	0.0055	0.0123	0.67^a^	0.004	0.0145
N2,N2-dimethylguanosine	0.82	0.0734	0.0519	0.80^a^	0.0205	0.0316

^a^*P* < 0.05.

Little has been reported on glucagon’s effects on urine AA concentrations and renal AA handling. Most of the known renal effects of glucagon are related to electrolyte handling, with a prominent role speculated for potassium homeostasis (Bankir *et al.* 2016). However, several reports support a role for glucagon in renal solute absorption, which may include AAs. One study found that proximal renal tubular cells expressed the glucagon receptor and that glucagon stimulated tubular glucose reabsorption (Marks *et al.* 2003). Another study measured urine AAs by total nitrogen and found there was no difference in urine disposition in response to a glucagon infusion (Boden *et al.* 1990). These data are similar to our study, where overall urinary excretion of AAs remained relatively stable despite lower plasma AA concentrations. In contrast, another study found increased urinary nitrogen excretion, which was attributed to increased ureagenesis (Fitzpatrick *et al.* 1977). Additionally, a case report documented increased urinary AA clearance in a patient with glucagonoma (Almdal *et al.* 1990), which may suggest that longer periods of glucagon exposure are required to result in demonstrative aminoaciduria.

The discrepancy between reduced plasma concentrations of AA and the apparent physiological adaptation for renal AA loss poses a challenge to interpretation. However, this paradoxical scenario might stem from the maladaptive consequences of exposure to supraphysiological levels of glucagon. Notably, glucagon has been shown to enhance AA transporter activity in the liver in a sodium-dependent manner (Lim *et al.* 1999). The observed greater proportional loss of AAs in urine suggests a reduction in tubular reabsorption, indicating that glucagon may exert tissue-specific effects on AA metabolism. Additionally, our *in vivo* approach opens the possibility of observing glucagon’s indirect effects on AA homeostasis. Thus, putative effects on renal AA transport could be mediated through other hormones modulated by glucagon.

An unexpected finding in our study was increased urinary 3-methylhistidine in response to the high-dose glucagon CRI relative to baseline levels. Increased urine concentrations of this AA are generally considered a marker of muscle catabolism; however, skeletal muscle does not express the glucagon receptor (Papatheodorou *et al.* 2020). Therefore, if increased urinary 3-methylhistidine in our study is reflective of muscle catabolism, the mechanism is likely indirect. Alternatively, the gut is a site of a considerable amount of 3-methylhistidine (Millward & Bates 1983) and could be the source contributing to increased urinary excretion in our study. Several previous studies have not found evidence of enhanced muscle catabolism or increased urinary 3-methylhistidine in response to glucagon infusion. However, these studies used lower doses (3–10 ng/kg/min) and either somatostatin (to inhibit insulin release) or exogenous glucose (to stimulate insulin release) (Fitzpatrick *et al.* 1977, James *et al.* 2022).

Although our cohort was relatively small, it fell within the reported range for preclinical studies involving dogs (Smith *et al.* 2002). Nonetheless, it is important to note that our cohort comprised a homogeneous group of dogs, which could limit the direct applicability of our findings to more diverse populations. To address this limitation, further investigations across different breeds are warranted to validate our results. For logistical expediency, we opted to use pre-infusion data as control data for each dog instead of employing a sham infusion. While this approach may raise concerns, it is worth noting that the negligible impact of a placebo saline infusion in humans, coupled with similar metabolic responses to exogenous glucagon, suggests that the utility of such a control may be limited. Moreover, our decision was supported by prior canine and cross-species data aligning with our observations.

As numerous studies have been conducted measuring AA concentrations in plasma in response to exogenous glucagon (Bromer & Chance 1969, Landau & Lugibihl 1969, Liljenquist *et al.* 1981, Boden *et al.* 1984, 1990, Couet *et al.* 1990, Flakoll *et al.* 1994), we did not quantitatively measure them in this study. Thus, our plasma AA measurements were semi-quantitative, while urine AA measurements were quantitative, prohibiting direct comparisons between the two compartments. Future investigations employing aligned quantitative methodologies designed to compare fluxes in plasma vs urine AA concentrations may yield deeper insights than our current study. Lastly, it is crucial to note that our study assessed the effects of a relatively brief period of elevated glucagon levels, which might not fully capture the alterations associated with prolonged hyperglucagonemia seen in chronic disease states.

Exogenous glucagon significantly alters the canine plasma metabolome independently of glucose levels. These data aid in our understanding of glucagon physiology in the dog which will inform our understanding of the pathophysiology of canine disorders involving glucagon, and where therapeutic targets, such as glucagon-like peptide agonists, will alter glucagon levels. Additionally, these data are an essential contribution to maintaining the dog as a translational model for studying glucagon biology. A novel finding was stable or increased urinary AA concentrations in the face of plasma AA reductions that suggest glucagon may affect renal tubular AA excretion and warrants further investigation.

## Supplementary Materials

Supplementary File 1

Supplementary Table 1. Significant metabolites detected between pre-infusion (PRE) and post-infusion (POST) metabolomes after 6-hour low-dose (LO) and high dose (HI) glucagon infusions in 5 dogs.

Supplementary Figure 1

Supplementary Figure 2

## Declaration of interest

The authors declare that the research was conducted in the absence of any commercial or financial relationships that could be construed as a potential conflict of interest.

## Funding

Cornell Universityhttp://dx.doi.org/10.13039/100007231 internal discretionary funds supported this study.

## Data availability statement

The raw data supporting the conclusions of this article, if not available within the article and its Supplementary Materials, will be made available by the authors, without undue reservation.

## Author contribution statement

JPL, MM, RWC, and BPC conceived of the study and conducted the data analyses. JPL, MM, RWC, CEF, LLB, SAP, and BM conducted the experiments. All authors contributed to writing the manuscript.
