# Supplementary material for: Glucagon infusion alters the circulating metabolome and urine amino acid excretion in dogs
Source: J Endocrinol. 2024 Jun 27;262(2):e240051. doi: 10.1530/JOE-24-0051 (PMC11301426; doi:10.1530/JOE-24-0051)
Supplement: Supplementary Figure 2 [file supplementary_figure_2.pdf]

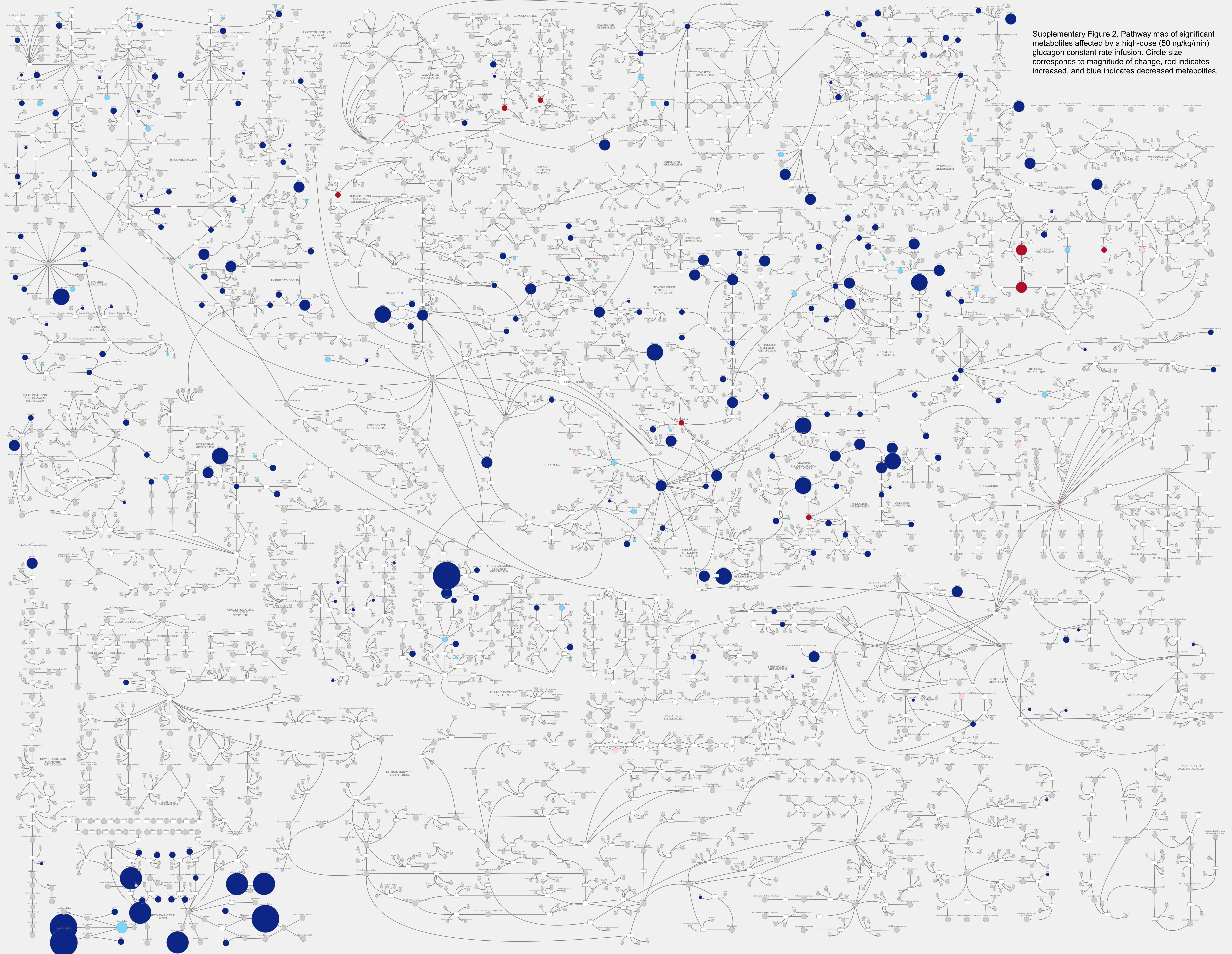

Supplementary Figure 2. Pathway map of significant metabolites affected by a high-dose (50 ng/kg/min) glucagon constant rate infusion. Circle size corresponds to magnitude of change, red indicates increased, and blue indicates decreased metabolites.
